# Twin-twin transfusion syndrome screening and diagnosis in the United States: A triangulation design of patient experiences

**DOI:** 10.1371/journal.pone.0200087

**Published:** 2018-07-05

**Authors:** Rebecca Fischbein, Lauren Nicholas, Julie Aultman, Kristin Baughman, Lynn Falletta

**Affiliations:** 1 Department of Family and Community Medicine, Northeast Ohio Medical University, Rootstown, Ohio, United States of America; 2 D’Youville College, Buffalo, New York, United States of America; 3 College of Public Health, Kent State University, Kent, Ohio, United States of America; Federal University of Sergipe, BRAZIL

## Abstract

**Objective:**

Using patient-reported experiences, this study: 1) quantitatively evaluated TTTS screening trends, 2) examined screening and diagnostic experiences using a mixed methods approach, and 3) determined gaps in clinical care experiences.

**Design:**

This was a cross-sectional study. Data was collected using a self-report, retrospective survey. A triangulation design was used to validate quantitative survey data with thematically analyzed qualitative data.

**Setting:**

Participants were recruited through social media and national foundations and completed the survey online.

**Participants:**

Participants were 312 women who completed a TTTS pregnancy in the United States, representing the largest survey of participants who have experienced TTTS.

**Methods:**

Descriptive statistics and bivariate analyses were conducted. Multivariate logistic regression examined predictors of ultrasound frequency. Qualitative data were initially coded by hand and checked using qualitative software.

**Results:**

The percentages of participants reporting guideline recommended screening, including identification of pregnancy type by gestational week 13 and timely receipt of ultrasounds, increased over time. However, 44.6% of participants diagnosed in recent years (2014 and later), reported that prior to TTTS diagnosis, they did not receive biweekly or more frequent ultrasounds. Three patient-reported provider practices were related to receiving ultrasounds at the recommended frequency: (1) determining MCDA status prior to gestational week 14, (2) providing participants with early warnings about the risk of TTTS to their pregnancies after MCDA status had been determined, and (3) referring participants to a Maternal-Fetal Medicine Specialist after MCDA identification, as validated by qualitative data. Our qualitative data revealed gaps in effective clinical care experiences among OB/GYN and specialist providers.

**Conclusion:**

These findings indicate screening and diagnosis for TTTS, as reported by patients, is improving in the United States; however, further efforts are required to ensure all patients receive appropriate screening, education and a team-based approach to comprehensive and supportive clinical care.

## Introduction

Twin-twin transfusion syndrome (TTTS) complicates 15% of monochorionic-diamniotic (MCDA) identical twin pregnancies [1]. TTTS carries a high risk of morbidity and mortality for fetuses but can be diagnosed with ultrasound and characteristically presents as an abundance of amniotic fluid for one fetus and lack of fluid for the other [2]. When left untreated, TTTS is 80–100% fatal; however, the increased use of interventions such as laser therapy has resulted in significantly improved survival rates for both twins (69.5%) and at least one twin (89.5%) [3]. TTTS treatments such as laser therapy have been shown most effective when performed in earlier stages of disease progression [4].

In 2013, the Society for Maternal Fetal Medicine published clinical recommendations for optimal MCDA management, suggesting: 1) early ultrasound examination at 10–13 weeks of gestation to determine shared placental status (monochorionicity), and 2) biweekly ultrasounds starting at 16 weeks of gestation and continuing through delivery [2]. Biweekly ultrasounds are critical as ultrasound frequency less often than every two weeks is associated with more advanced TTTS stage at diagnosis [5]. Subsequent to these recommendations, the American Colleges of Obstetrics and Gynecologists have also published ultrasound screening guidelines that indicate that late first trimester/early second trimester screening is indicated to assess for chorionicity [6]. However, despite recommendations, little is known about the timeliness of United States patients’ ultrasound screenings and their experiences related to MCDA identification and TTTS screening and diagnosis. Supplemental to this inquiry, the authors also sought to understand if women are being warned about the risks of TTTS, or are receiving referrals to Maternal-Fetal Medicine Specialists (MFM Specialists) after monochorionicity identification. Finally, we sought to understand clinical care experiences, particularly as related to screening and diagnosis, of women who had a pregnancy with TTTS.

Lack of data about patients’ screening and diagnostic experiences may be partly due to information gaps about patients prior to presenting at fetal treatment centers [7], and the difficulty in obtaining a large number of medical records for a rare disease such as TTTS. Given the relative rareness of TTTS and the lack of interoperability among electronic health record systems across the United States, it is impracticable to obtain national data about healthcare practices and screening experiences in this manner. As a first step in understanding the care patients receive, this study aimed to evaluate patient-reported TTTS screening experiences in the United States using a mixed methods approach.

## Materials and methods

A retrospective, online survey about TTTS pregnancy experiences was targeted towards women, over the age of 18, living in the United States at the time of pregnancy who completed a TTTS pregnancy. Responses were gathered over a 4-month period in 2016. Participants were recruited through social media groups devoted to TTTS, identical twins, MCDA pregnancies and twins, twin loss, and multiples loss, and through national foundations, including the Twin to Twin Transfusion Syndrome Foundation, Fetal Health Foundation, and Mother of Multiples of America/Mother of Twins. Participants were fully informed that the mixed-methods survey was anonymous. The process of informed consent required participants to review a detailed consent document prior to participating in the survey, and included contact information for the Principal Investigator. They were also provided with contact information for crisis services in the event of a mental or behavioral health crisis during or after participation in the survey. This research was approved by the institutional review boards of Kent State University (protocol number 16–169), D’Youville College and Northeast Ohio Medical University (protocol number 17–010).

### Sample

Three hundred and ninety-four women participated in the survey. Twenty participants did not meet inclusion criteria, reducing the sample to 374 respondents. To reduce the possibility of recall bias, the sample was further reduced to 312 to include only women who had been diagnosed with a TTTS pregnancy within the past ten years (2006 to 2016). The respondents were predominately white (89.7%), with a minimum of some college education (90.3%), married (82.1%), and at least $60,000 in annual family income (66.0%). The average participant age during the TTTS pregnancy was 30.3 and most had private insurance (70.6%).

### Measures

The online survey asked participants to respond to questions related to their TTTS pregnancy in the following domains:

#### Demographics

Demographic questions included: age, race, marital status, annual family income, insurance status, education, year of TTTS diagnosis.

#### Screening and diagnostic experiences

A variable was created to indicate if the TTTS diagnosis occurred during or after 2014, capturing the impact of clinical recommendations published in 2013. Questions related to patient-reported diagnostic experiences in the current study included: 1) gestational week MCDA was identified, 2) type of healthcare provider who determined MCDA pregnancy, 3) warnings about TTTS, 4) referrals to MFM Specialists after MCDA identification, 5) frequency of ultrasounds prior to TTTS diagnosis, 6) treatment for other prenatal conditions prior to TTTS diagnosis, and 7) receipt of routine prenatal care with obstetrician prior to TTTS diagnosis. Gestational week of MCDA determination was dichotomized with 1 indicating MCDA determination before or during gestational week 13 and 0 denoting after week 13. Ultrasound frequency was dichotomized with 1 representing biweekly or more frequent ultrasounds and 0 as less frequent. Pregnancy outcome was assessed (no survivors, single survivor, double survivors) and included in this analysis to control for the possibility that outcome could have influenced participant recall. Open ended questions about patient experiences included: “Do you feel as if you received the best possible care you could have by your primary obstetrician? Please describe, in detail, why yes or no”, “Do you feel as if you received the best possible care you could have by your perinatologist/maternal fetal medicine (MFM) specialist? Please describe, in detail, why yes or no.”, “Did you ever feel the need to advocate for additional care to your primary obstetrician (or other primary care providers)? If you answered yes, please describe how you advocated and why”, if participants answered yes to the previous question “Did your primary obstetrician (or other primary care providers) listen to your concerns and act accordingly?” and “Please give any comments you feel are vital regarding your TTTS experience that were not collected within this survey”.

### Data analysis

The research team separated quantitative data from qualitative data to conduct analyses, producing individual quantitative and qualitative results. The weight of the survey data rests with the quantitative data (designated by QUAN), and therefore is validated with the qualitative results (designated by qual) using combined (QUAN + qual) interpretation of those results. Investigators conducting the initial quantitative analysis did not conduct the qualitative analysis to reduce potential bias; however, investigators came together to discuss and interpret the combined (QUAN + qual) data. This triangulation design, which validates quantitative data with complementary qualitative data, is a common approach for mixed-methods survey analysis and interpretation [8], particularly when a quantitative survey includes a few open-ended qualitative questions. This method also permits our research team to expand our quantitative results with qualitative information that allows us to interpret future research needs, develop policy, and inform medical education. Due to the limited qualitative data, thematic analysis was conducted to identify themes that emerged in the data.

Quantitative data were analyzed using Stata 14. Bivariate analyses, including chi-square and t-tests, and a multivariate logistic regression predicting ultrasound frequency were conducted. Qualitative data were coded by hand and checked using Atlas.ti (7^th^ edition).

## Results

### Quantitative findings

**Fig 1:**
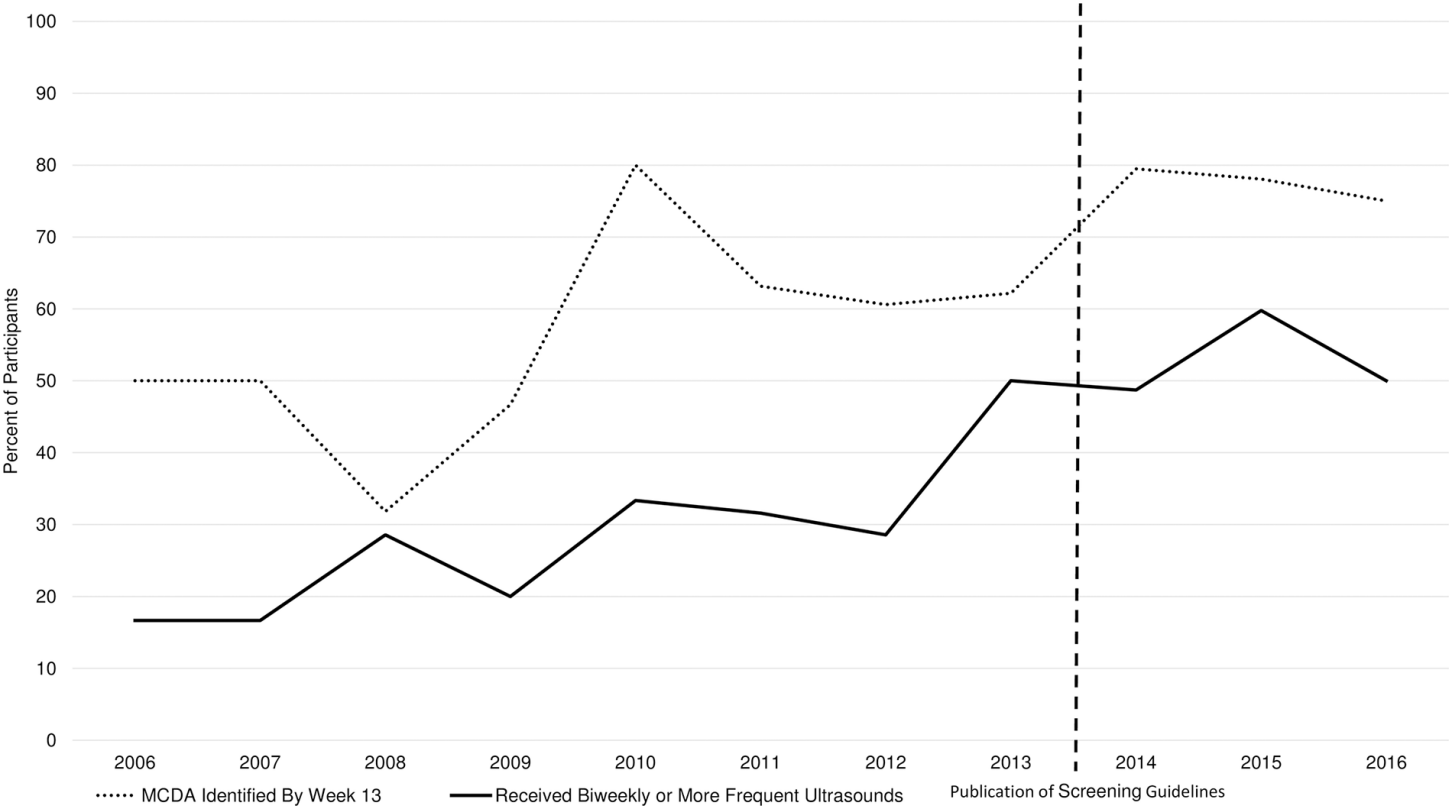
Patient-reported trends in MCDA identification and screening for TTTS.

Fig 1 demonstrates diagnosis and screening trends as reported by participants. Over time, increasing percentages of participants reported having monochorionicity established by week 13. Additionally, the percentage of participants reporting receipt of biweekly or more frequent ultrasounds prior to TTTS diagnosis increased over time, particularly after publication of recommendations in 2013.

To further explore how the dissemination of TTTS screening recommendations in 2013 may have impacted patient-reported screening experiences, comparisons were made before and after 2014 (Table 1). There were no significant differences in the number of participants receiving routine prenatal care prior to TTTS diagnosis, number of participants receiving advanced prenatal care due to pregnancy complications not related to TTTS, or the distribution of the type of provider who first determined monochorionicity. Participants diagnosed with TTTS in 2014 and later were marginally more likely to have been informed about the risk of TTTS after monochorionicity identification (62.2%) than those diagnosed in prior years (52.4%). Participants diagnosed with TTTS in later years were also more likely to report referral to a MFM Specialist after monochorionicity identification (72.3%) than those diagnosed between years 2006–2013 (60.4%). Similarly, participants with more recent diagnoses were more likely to have monochorionicity established during or before gestational week 13 (75.7%) than those diagnosed with TTTS in earlier years (60.4%). In addition, the number of participants who reported receiving biweekly or more frequent ultrasounds prior to their TTTS diagnosis significantly increased after publication of screening recommendations from 31.7% to 52.7%, respectively. Nearly half (44.6%) of all participants diagnosed with TTTS in 2014 and later indicated they were not receiving biweekly or more frequent ultrasounds prior to their TTTS diagnosis.

**Table 1:** Patient-reported physician practices before and after 2014 (n = 312).

	Before 2014 n (%)	2014 and After n (%)	Statistic (p)
Year of TTTS Diagnosis (Median)	2011	2015	
Prenatal Treatment Due to Complications Prior to TTTS Diagnosis			
Yes	23 (14.0)	13 (8.8)	χ^2^(1) = 0.01 (0.91)
No	137 (83.5)	131 (88.5)	
Missing	4 (2.4)	4 (2.7)	
Receipt of Routine Prenatal Care Prior to Diagnosis			
Yes	153 (93.3)	140 (94.6)	χ^2^(1) = 2.08 (0.15)
No	3 (1.9)	3 (2.0)	
Missing	8 (4.9)	5 (3.4)	
Healthcare Provider Who First Identified MCDA Status			
MFM Specialist	51 (31.1)	35 (23.6)	χ^2^(4) = 6.53 (0.16)
Primary Care Provider(s)	70 (42.7)	57 (38.5)	
Reproductive Endocrinologist	7 (4.3)	14 (9.5)	
Ultrasound Technician	29 (17.7)	34 (23.0)	
Participant never told MCDA status	4 (2.4)	2 (1.4)	
Missing	3 (1.8)	6 (4.1)	
Informed About Risk of TTTS After MCDA Identification			
Yes	86 (52.4)	92 (62.2)	χ^2^(2) = 5.38 (0.07)
No	68 (41.5)	51 (34.5)	
I don’t know	6 (3.7)	1 (0.7)	
Missing	4 (2.4)	4 (2.7)	
MCDA Identification During or Before 13 Weeks			
During or Before	90 (54.9)	112 (75.7)	χ^2^(1) = 15.24 (0.00)
After	69 (42.1)	32 (21.6)	
Missing	5 (3.0)	4 (2.7)	
Patient Referred to MFM Specialist After MCDA Identification			
Yes	99 (60.4)	107 (72.3)	χ^2^(3) = 5.94 (0.05)
No	60 (36.6)	37 (25.0)	
I don’t know	1 (1.0)	4 (2.7)	
Missing	5 (3.0)	4 (2.7)	
Biweekly or More Frequent Ultrasounds Prior to TTTS Diagnosis			
Yes	52 (31.7)	78 (52.7)	χ^2^(3) = 14.86 (0.00)
No	109 (66.5)	66 (44.6)	
Missing	3 (1.8)	4 (2.7)	

**TTTS**: Twin-twin transfusion syndrome, **MCDA**: Monochorionic Diamniotic

Bivariate analyses explored factors related to the receipt of biweekly or more frequent ultrasounds prior to TTTS diagnosis. This analysis was performed using participants diagnosed with TTTS in the years following the publication of the 2013 recommendations. Participant characteristics including annual income, insurance and education levels were associated with receipt of timely ultrasounds. Pregnancy outcome was not related to reported ultrasound frequency indicating that participant recall of ultrasound frequency was likely not biased based on outcome. Three screening and diagnostic factors were related to ultrasound frequency: (1) monochorionicity established during or before gestational week 13 (χ^2^ = 9.02, p<0.01); (2) being informed about the risk of TTTS after monochorionicity determination (χ^2^ = 10.74, p = 0.01); and (3) referral to a MFM Specialist after monochorionicity identification (χ^2^ = 12.00, p < .01).

A multivariate logistic regression identified factors predicting if biweekly or more frequent ultrasounds were received prior to TTTS diagnosis (Table 2). The analysis included all variables established through bivariate analyses to be significantly related to frequency of ultrasounds prior to TTTS diagnosis. Participants informed about the risks of TTTS upon monochorionicity identification had increased odds of receiving biweekly or more frequent ultrasounds (OR 2.82, CI 1.21, 6.56) compared to those who were not warned about risks. In addition, the odds of receiving timely ultrasounds were 4.45 (CI 1.66, 11.93) times higher for those referred to a MFM Specialist upon monochorionicity establishment compared to those who were not referred at that time. Lastly, individuals with monochorionicity determined during or prior to gestational week 13 had increased odds of receiving biweekly or more frequent ultrasounds (OR 4.95, CI 1.72,11.96) compared to those who did not.

**Table 2:** Multivariate logistic regression predicting biweekly TTTS screening ultrasounds among patients diagnosed 2014 and after (n = 135).

	Adjusted Odds Ratio^a^ (p value)	95% Confidence Interval	
		5%	95%
**Participant Demographics**			
Annual Family Income^b^			
Less than $60,000 (Reference)	-	-	-
$60,000 to $99,999	1.53 (0.49)	0.46	5.07
Over $100,000	1.55 (0.48)	0.46	5.21
Insurance			
Private (Reference)	-	-	-
Public	0.40 (0.19)	0.10	1.58
Combination	0.44 (0.57)	0.27	7.19
Tricare	0.84 (0.86)	0.14	5.30
Other^c^	0.45 (0.60)	0.23	8.72
Education^d^			
High School Degree or Less (Reference)	-	-	-
Some or More College	3.42 (0.19)	0.55	21.40
**Screening and Diagnostic Experiences**			
Informed About Risk of TTTS After MCDA Identification^e^			
No (Reference)	-	-	-
Yes	2.82 (0.02)	1.21	6.56
Patient Referred to MFM Specialist After MCDA Identification^f^			
No (Reference)	-	-	-
Yes	4.45 (0.00)	1.66	11.93
MCDA Identification During or Before 13 Weeks^g^			
During or Before	4.95 (0.00)	1.72	11.96
After (Reference)	-	-	-

**MCDA**: Monochorionic Diamniotic, **TTTS**: Twin-twin transfusion syndrome

^a^Higher Adjusted Odds Ratios represent increased likelihood for biweekly or more frequent ultrasounds relative to reference group.

^b^Annual Family Income was coded 1 = Less than $60,000, 2 = $60,000-$99,999, 3 = $100,000 and over

^c^Includes “I did not have health insurance” and “I don’t know”

^d^Education was coded 0 = High School Degree or Less, 1 = Some or More College

^e^Informed About Risk of TTTS After MCDA Identification was coded 0 = No, 1 = Yes, 2 = I Don’t Know

^f^Patient Referred to MFM Specialist After MCDA Identification was coded 0 = No, 1 = Yes.

^g^MCDA Identification During or Before 13 Weeks was coded 0 = After 14 weeks, 1 = During or Before 13 weeks

### Qualitative findings

Two hundred and forty-five participants responded to the questions regarding perceptions of care received by primary care obstetrician and perinatologist/MFM specialist. Responses were categorized into those who indicated they: received the best possible care (52.7%, n = 129), did not receive the best possible care (37.14%, n = 91) and received mixed quality of care (10.2%, n = 25). Table 3 provides themes based on positive, negative or mixed responses.

**Table 3:** Respondents’ descriptions of care received by providers.

Open-ended questions	Number of Respondents	Themes based on Positive, Negative, or Mixed Responses	Example Quotations
Do you feel as if you received the best possible care you could have by your primary obstetrician? Please describe, in detail, why yes or no.	129 responded **POSITIVELY** (received best possible care)	Knowledgeable of TTTS and other difficult pregnancies Communicates well and provides thorough information Makes appropriate referrals to MFM or other specialist in a timely manner Provides comprehensive and continuous care with multiple ultrasounds and prenatal visits. Works collaboratively with MFM or other specialist Continues the therapeutic relationship even if most care is delivered by MFM Supportive and recognizes needs of patient Takes patient reports seriously and involves patient in her care	“I went to my OB with my concerns about sudden weigh gain and swelling. 15 lbs in a week. She immediately referred me to a MFM doctor. Unfortunately I started contracting and had to go to the ER before going to the specialist. I was airlifted to the University [hospital]…where Dr. [X] diagnosed me with TTS and performed the laser ablation surgery. I will forever be grateful for [her] saving my boys.”
			“He was very supportive and helped guide decisions. Felt that he and MFM had differing options in regard to recommendation for termination and I thought he (OB) was helpful in outlining the pros and cons. Obviously chose to NOT terminate but felt like he helped support that decision.”
			“I saw the MFM until the babies were stable after surgery. My OB was very interested and invested in my pregnancy. We decided many details (delivery, gestation length) in length. She made me feel very involved and made sure I saw the MFM right away.”
			“I was provided the option to 100% switch my care to the MFM Dr. I chose not to, and to work with both doctors. I love my primary OB-GYN. She is part of the same practice as the MFM and they worked together throughout my pregnancy. She was very knowledgeable as well
	91 responded **NEGATIVELY** (did not receive best possible care)	Lack of OB understanding (incompetent, ill-informed) of the disease Lack of emotional support for patient and/or family Poor bedside manner: negative responses to patients such as belittlement; patients told “not to worry”; Referral to MFM or other specialist took too long or not at all More ultrasounds Lack of responsiveness to mother’s questions and needs, including her instincts about an abnormal pregnancy and pain levels. Poor disclosure or lack of disclosure of risks of TTTS pregnancy General lack of information about TTTS post-diagnosis	“I had absolutely horrendous care. I was told all my concerns and side effects were just part of being pregnant. I was belittled and even after several hospitalizations I was refused MFM care until we discovered TTTS…poor medical care killed my daughters.”
			“No. She didn’t see to know or care the importance of monitoring in a pregnancy like this. She didn’t monitor after finding out there were twins. She didn’t increase ultrasound frequency.”
			“I got told it is a regular pregnancy and not worry till’ they told me to. And not to research TTTS because that only added stress.”
			“They were completely unaware of TTTS or what to watch for in my pregnancy, etc.”
			“She did not refer me to a specialist once she knew I was having identical twins. She did not recognize the symptoms I was having as symptoms of TTTS. She knew about TTTS but didn’t explain it to us once we knew we were having ID twins. She just said there is a really rare thing that can happen but don’t even worry about it.”
	25 had **MIXED** responses (good and poor care)	Combinations of the above positive and negative themes	“I’m still trying to understand how the fact I was carrying twins was missed even after multiple ultrasounds. However, when I experienced maternal symptoms (excessive weight gain, tightness) my OB took my concerns seriously and immediately referred me to an MFM.”
			“My primary OB had no understanding of choronicity (sic) and confused me greatly. I was referred to MFM almost immediately however and was very happy with my care.”
			“He was great, but knew nothing about TTTS but the basics. I should have been referred to MFM early on. He never referred me. Went for 19 week ultrasound and found out TTTS. MFM was on site at ultrasound office. Once confirmed, he saw me twice weekly.”
			“She immediately transferred me to a specialist when I was diagnosed, but I think I should have been monitored more closely before my diagnosis. I had an ultrasound at 8 weeks to date the pregnancy and then not another one until 19 weeks when TTTS was diagnosed.”
“Do you feel as if you received the best possible care you could have by your perinatologist/maternal fetal medicine (MFM) specialist? Please describe, in detail, why yes or no	171 responded **POSITIVELY** (received best possible care)	Speed of treatment, particular surgical treatment; recognized urgency of care Knowledgeable Keeping close watch/ monitoring Attentive to patient and family needs Respectful Offered Options; allowed patients to participate in decision making Offered complete information; disclosure of risks Offered hope Did not push termination/abortion	“Yes I was offered treatment plans, informed of all complications, and MOST IMPORTANTLY they talked to us with respect. They described all medical terms and never shied away from being absolutely honest while still offering hope!”
			“Yes. We had 2 very good, well-informed doctors that saw us and gave us a lot of information. They were very proactive and gave us a lot of information at every appointment. They informed us about laser ablation at our first appointment at 14 weeks. They did not push selective reduction or termination.”
			“While we dealt with numerous MFM doctors throughout our TTTS experience they were all very professional and caring. They always wanted the best well-being for myself and our babies.”
			“YES! I named one of my boys after him.”
	34 responded **NEGATIVELY** (did not receive best possible care)	Poor bedside manner Difficult to communicate with/lack of complete information Lack of emotional support/hope Lack of comprehensive and continuous patient care Less frequent visits/lack of attentiveness Lack of extensive diagnostic care	“No. He did tell me about the possibility of TTTS but didn’t inform me of any of the signs or symptoms to watch for.”
			“My MFM told me to abort babies and that laser was not an option for me. She was WRONG. I had surgery after I found a clinic in WA state on my own, and both babies survived and are healthy.”
			“My twins actually went undiagnosed until delivery. If they would have done the more extensive Doppler’s, I believe they would have caught it earlier on.”
			“No. I barely saw them before the surgery then didn’t see them again. I couldn’t even tell you their name.”
	30 had **MIXED** responses (good and poor care)	Combinations of the above positive and negative themes	“She was very direct about my diagnosis and suggested that I see a specialist and she gave me two options. Then I left and had to research those options and make an appointment fast as the TTTS was progressing quickly. I also had to make reservations to fly to the destination where a specialist could do the laser ablation surgery, and I was offered no help in terms of financial assistance or referred to the Twin to Twin Transfusion Syndrome Foundation for help. I think she did her job for which I am grateful but did the minimum.”
			“Medically yes. He was excellent. However, after he was done with his part I never saw him again. I work in the same hospital and when I see him he never acknowledges me. It would be nice to have a little more emotional support from him after experiencing something so traumatic under his care.”
			“Excellent clinician but hard to communicate/open discussion with. But he got me to the people who communicated better and supported me.”

Themes that validate the quantitative findings include the importance of early referral to MFM, provision of comprehensive care through multiple ultrasounds and prenatal visits, and communication of information to patients:

“I am very lucky that my OB was knowledgeable about TTTS and referred me to a MFM right away. He also did an ultrasound at my first prenatal appointment and was able to determine modi right away. He also communicated well with the MFM and followed their recommendations for continued care.”

Likewise, patients who did not receive the best possible care noted the lack of these clinical practices:

“I feel as though I should have had more ultrasounds leading up to the diagnosis. I had one at 16 weeks then not again until 19 when I was diagnosed.”

Positive and negative themes that that emerged outside the scope of the quantitative analysis included the importance of the therapeutic relationship between the primary obstetrician and patient, which includes open communication and support, as well as the importance of listening to the patient’s concerns that something was not right with the pregnancy:

“…when I described how uncomfortable I was and how I was in pain, I was laughed at and made to think I was being over dramatic (this was my 3rd pregnancy and my first 2 were very pleasant).”

Most participants (70.9%, n = 171) indicated their perinatologists/MFM Specialists provided the best possible care. Themes focused on the specialists’ recognition of the urgency for care: “Right away upon meeting mentioned the need to monitor for TTTS. Then when TTTS appeared referred me to a fetal surgeon within days.”

The therapeutic relationship between the specialist, obstetrician, and the patient was emphasized in positive care experiences, particularly in terms of *team-based* communication, respect, and attentiveness. Conversely, participants who reported they did not receive the best possible care (13.9%, n = 34) highlighted the lack of these components of the patient-physician relationship, as well as the lack of urgency of care (see Table 3). Further, one patient reported practice policy prevented her from receiving more frequent visits:

“Their policy was one visit every 4 weeks. At my final appointment I was 18 weeks pregnant and there was already an almost 20% size discordance and despite that and my concerns (which were dismissed), I wasn’t scheduled for another appointment for almost 5 weeks.”

One hundred and seventy-three participants reported how they advocated for care from their primary care obstetrician (Table 4). Emerging themes focused on seeking additional knowledge through self-education, second opinions, or expert advice. Other participants reported having to demand or beg for additional services such as additional ultrasounds or interventions: “I did personal research and begged for interventions but was denied.”

**Table 4:** Respondents’ descriptions of self-advocacy and recommendations.

Open-ended questions	Number of participants	Themes: How respondents advocated for themselves.	Example Quotations
Did you ever feel the need to advocate for additional care to your primary obstetrician (or other primary care providers)? If you answered yes, please describe how you advocated and why.	173	Personal research/educated oneself Talked to experts/asked to see experts Sought a second opinion Begged Demanded/insisted on additional tests or better care Questioned Regret not acting…wished they did more Found new doctor Tried out alternative care without EBM/significant research	“I did personal research and begged for interventions but was denied.”
			“I had to ask for weekly ultrasounds for cervical checks and for them to stay in contact with [OB doctor].”
			“I demanded to be see more often. I asked loads of questions. I received multiple opinions and made my own decisions.”
			“Had to advocate for MCA Doppler. Had to educate myself on TTTS, for the most part.”
			“Asking questions, not accepting the recommendation to terminate, asking for second opinion.”
			“I put myself on a high protein diet. My care team said there was no evidence it did anything. In my mind it didn’t matter if it might help. I was going to try.
.Please give any comments you feel are vital regarding your TTTS experience that were not collected within this survey	129	Knowledge/education/awareness of OBs and health professionals Grief counseling/support groups Continuous monitoring/ultrasounds Look into alternative practices and expand research (including patient -centered research) Access to care Access to information/patient education Better communication/listening to patients Trusting parent/patient Recognizing patient fear and need for emotional support	“I went from biweekly ultrasounds and appointment to weekly. Should they have started as weekly and then been upgraded to biweekly? I don’t know. Somehow this needs to be figured out. To this day I still feel that I did something wrong to lose my babies.”
			“…parents should be offered grief counseling after the death of their child/children. It should be mandatory (the formal offer by doctors that is).”
			“I feel that protein therapy should have been explored as an option from my medical team…I think there needs to be more awareness and education about the disease. I feel it is not as rare as many perceive it to be.”
			“I feel as more research and ALL OBs should be educated thoroughly on TTTS and all women with mono di twins should be carefully monitored for TTTS with weekly ultrasounds.”
			“I did not know that I should have been asking to see a maternal fetal medicine specialist. I was not informed about TTTS. I trusted my primary OB to guide me and I feel that she failed me.”
			“I believe that doctors need to be willing to listen to women more. They may have all the data and experience in the world, but mothers know their own bodies and their babies better. I think my outcome may have been different if the doctors placed as much trust in me as I did in them.”
			“It was very scary to be told one baby might live. I think better ways of handling a mother who [is] scared to death should be better handled.”

One hundred and twenty-nine participants responded to the final open-ended question of the survey, which asked participants to provide additional thoughts or comments (Table 4). The majority of these comments consisted of recommendations for improved care of TTTS pregnancies. Suggestions focused on increased education and awareness of TTTS by primary obstetricians and patients, increased access to care, improved emotional support, and policy changes surrounding screening: “I think it should be mandatory for all multiple pregnancies to get weekly ultrasounds, and they should immediately be recommended to an MFM.”

## Discussion

In this study we found that participants diagnosed after the publication of screening recommendations reported higher rates of biweekly or more frequent ultrasounds and monochorionicity identification prior to gestational week 14. Further, participants with more recent pregnancies also reported increased likelihood of referral to MFM Specialists as well as warnings from healthcare providers about the risk of TTTS. These findings suggest that implementation of recommended TTTS screening procedures, as well as awareness of risks related to MCDA pregnancies, may be increasing in the United States. However, in the years following the publication of screening recommendations, only 52.7% of participants reported receiving ultrasounds at, or greater than, the recommended frequency.

Our qualitative findings confirm the women’s quantitative answers in terms of the importance of frequent ultrasounds, referral to MFM upon MCDA diagnosis and communication of risks. Qualitative results also help provide context to interpret the quantitative findings. For example, the lack of prenatal visits and ultrasounds may be perceived by patients as the result of hospital or practice policy, but may in fact be a result of reimbursement issues or current practice guidelines.

Current recommendations suggest that MCDA status be identified by gestational week 13 followed by biweekly ultrasounds [2]; however, practice guidelines also suggest that unless otherwise indicated, a patient receiving a single ultrasound examination during pregnancy should receive it around weeks 18–22[6]. In addition, there are no current clinical guidelines in the US recommending that MCDA pregnancies be referred to MFM specialists, yet our research suggests that referral to MFM plays an important role both in timely receipt of ultrasounds and in patient perceptions of care. Considering the high risk nature of MCDA pregnancies [9,10], it may be advisable for healthcare providers to regularly refer patients with MCDA pregnancies to MFM Specialists.

Beyond the scope of the quantitative research, the qualitative findings highlight the importance of the team-based relationship between the patient, obstetrician, and specialist. Receiving continuity of care through a team approach comprised of both primary obstetrician and specialist was reported by participants who indicated they received high quality care. Team-based care teams have been demonstrated to be associated with patient satisfaction and improved outcomes [11,12].

In addition, these results emphasize the role of the therapeutic relationship between patient and healthcare provider. Feeling listened to, respected, supported and receiving ample information impacts patient perceptions of the care they received. Educating patients about the risks of TTTS has the potential to enhance patient empowerment related to disease management and to improve outcomes through several pathways. Patients may demonstrate increased adherence to recommendations, self-monitor more diligently, and increase utilization of health services when symptoms indicate an acute crisis and need for care [13]. Indeed, several review studies indicate that effective physician-patient disease communication is associated with improved health outcomes [14,15].

While this research is the first to report pre-TTTS screening experiences using a large sample of mothers who experienced TTTS, there are limitations to the present work. This study used social media for recruitment which likely missed underserved populations and possibly skewed our sample towards individuals who had better and/or worse TTTS experiences and outcomes. However, prior research supports the use of social media to recruit for the study of rare disease [16]. In addition, there was a lack of diversity among the participant population, despite research suggesting that there are limited racial differences among social media use [17]. This may indicate limited diversity within the composition of the social media groups. However, survey respondents possessed overwhelmingly favorable characteristics as they relate to the delivery of healthcare in the US: white, average to high income, high levels of education, married, and privately insured. Despite these protective factors that typically guard against poor healthcare quality [18] nearly half of all women diagnosed after the dissemination of TTTS screening recommendations reported lack of timely ultrasounds. This raises speculative concern for the level of care encountered by populations who traditionally experience health disparities, and further investigation is warranted.

Further, as a self-reported, retrospective survey, recall bias may have been a problem [19]. However, maternal recall obtained within four to six years of the prenatal experience has been found to be 89% accurate when compared to patient charts [20].

As reported by patients in our study, screening for TTTS may be improving in the United States. However, the need remains for all patients to receive adequate and timely TTTS screening. Our research also indicates that other physician practices such as early identification of monochorionicity, warnings about TTTS and referral to MFM specialist upon monochorionicity establishment are related to increased ultrasound frequency, ultimately improving patient care.

## Supporting information

S1 File TTTS patient survey. (DOCX)
