# Supplementary material for: Twin-twin transfusion syndrome screening and diagnosis in the United States: A triangulation design of patient experiences
Source: PLoS One. 2018 Jul 5;13(7):e0200087. doi: 10.1371/journal.pone.0200087 (PMC6033438; doi:10.1371/journal.pone.0200087)
Supplement: S1 File — (DOCX) [file pone.0200087.s001.docx]

In what year were you diagnosed with TTTS?

At the time of your TTTS diagnosis, how old were you?

Are you White, Black or African-American, Hispanic, American Indian or Alaskan Native, Asian, Native Hawaiian or other Pacific islander, or some other race?

- White (1)
- Black or African American (2)
- Hispanic (3)
- American Indian or Alaska Native (4)
- Asian (5)
- Native Hawaiian or Pacific Islander (6)
- Other (7)

At the time of your TTTS diagnosis, what was the highest level of school you had completed or the highest degree you had received?

- Less than high school degree (1)
- High school degree or equivalent (e.g., GED) (2)
- Some college but no degree (3)
- Associate degree (4)
- Bachelor degree (5)
- Graduate degree or higher (6)
- Other (please specify) (7) ____________________

At the time of your TTTS diagnosis, were you married, not married but co-habitating, widowed, divorced, separated, or never married?

- Married (1)
- Not married but co-habitating (2)
- Widowed (3)
- Divorced (4)
- Separated (5)
- Never married (6)

At the time of your TTTS diagnosis, in what state or U.S. territory did you live?

- Alabama (1)
- Arizona (2)
- Arkansas (3)
- California (4)
- Colorado (5)
- Connecticut (6)
- Delaware (7)
- District of Columbia (8)
- Florida (9)
- Georgia (10)
- Idaho (11)
- Illinois (12)
- Indiana (13)
- Iowa (14)
- Kansas (15)
- Kentucky (16)
- Louisiana (17)
- Maine (18)
- Maryland (19)
- Massachusetts (20)
- Michigan (21)
- Minnesota (22)
- Mississippi (23)
- Missouri (24)
- Montana (25)
- Nebraska (26)
- Nevada (27)
- New Hampshire (28)
- New Jersey (29)
- New Mexico (30)
- New York (31)
- North Carolina (32)
- North Dakota (33)
- Ohio (34)
- Oklahoma (35)
- Oregon (36)
- Pennsylvania (37)
- Rhode Island (38)
- South Carolina (39)
- South Dakota (40)
- Tennessee (41)
- Texas (42)
- Utah (43)
- Vermont (44)
- Virginia (45)
- Washington (46)
- West Virginia (47)
- Wisconsin (48)
- Wyoming (49)
- Puerto Rico (50)
- Alaska (51)
- Hawaii (52)
- I did not reside in the United States (53)

At the time of your TTTS diagnosis, what was your annual family income?

- Less than $19,999 (1)
- $20,000 - $39,999 (2)
- $40,000 - $59,999 (3)
- $60,000 - $79,999 (4)
- $80,000 - $99,999 (5)
- $100,000 - $119,999 (6)
- $120,000 - $139,999 (7)
- More than $140,000 (8)

At the time of your TTTS diagnosis, did you have private or public (state-funded) insurance?

- Private (1)
- Public (2)
- Combination (3)
- Tricare (4)
- I did not have health insurance (5)
- I don't know (6)
- Other (7) ____________________

In what gestational week did you confirm with a doctor that you were pregnant?

In what gestational week were you told that you were having monochorionic-diamniotic twins?

Who first told you were having monochorionic-diamniotic twins? (You may select more than one)

- Obstetrician (1)
- Perinatologist/Maternal Fetal Medicine (MFM) specialist (2)
- Nurse (3)
- Midwife (4)
- Nurse Practitioner (5)
- Physician Assistant (6)
- Ultrasound Tech (7)
- Reproductive endocrinologist/fertility specialist (8)
- Other (11) ____________________
- I don't know (9)
- I was not informed I was having monochorionic-diamniotic twins (10)

At this time were you told about the possibility of TTTS in monochorionic-diamniotic multiple pregnancies?

- Yes (1)
- No (2)
- I don't know (3)

At this time, when you were told you were carrying a monochorionic-diamniotic pregnancy, were you referred to a perinatologist or Maternal Fetal Medicine (MFM) Specialist?

- Yes (1)
- No (2)
- I don't know (4)

PRIOR TO your TTTS diagnosis, how frequently were you receiving ultrasounds?

- Multiple times per day (1)
- Daily (2)
- Multiple times per week (3)
- Weekly (4)
- Bi-weekly (5)
- Less than bi-weekly (6)
- I was not receiving regular ultrasounds - just one or two (7)
- I don't know (8)

PRIOR TO your TTTS diagnosis, were you being treated for any other prenatal complications?

- Yes (1)
- No (2)
- I don't know (3)

What prenatal complications were you being treated for (for example, gestational diabetes, hypertension, pre-eclampsia)?

In what gestational week were you first diagnosed with TTTS?

Did you experience any TTTS symptoms PRIOR TO the diagnosis?

- Yes (1)
- No (2)
- I don't know (3)

If you answered yes to the previous question, what symptoms did you experience? (You may select more than one)

- Swelling (1)
- Sudden weight gain (2)
- Contractions (3)
- Decreased fetal movement (4)
- Pain (5)
- Other (please specify) (6) ____________________

In which gestational week did these symptoms FIRST appear?

Did you share these symptoms with your healthcare provider?

- Yes (1)
- No (2)
- I don't know (3)

With which healthcare provider did you share your symptoms? (You may select more than one)

- Obstetrician (1)
- Midwife (2)
- Nurse (3)
- Midwife (4)
- Nurse Practitioner (5)
- Physician Assistant (6)
- Ultrasound Tech (7)
- Reproductive endocrinologist/fertility specialist (8)
- I don't know (9)

Do you believe your healthcare provider responded to your concerns appropriately?

- Yes. Please describe provider response (1) ____________________
- No. Please describe provider response (2) ____________________
- I don't know (3)

On the day you were first diagnosed with TTTS, what Stage were you diagnosed as? (You may select more than one)

- Stage I (1)
- Stage II (2)
- Stage III (3)
- Stage IV (4)
- Stage V (5)
- I don't remember (6)
- I was never told what Stage I was (7)
- Other (please specify) (8) ____________________

Up until your TTTS diagnosis, were you receiving routine prenatal care with an obstetrician?

- Yes (1)
- No (2)
- I don't know (3)

Who made your initial TTTS diagnosis? (You may select more than one)

- Obstetrician (1)
- Perinatologist/Maternal Fetal Medicine (MFM) specialist (2)
- Nurse (3)
- Midwife (4)
- Nurse Practitioner (5)
- Physician Assistant (6)
- Ultrasound Tech (7)
- Reproductive endocrinologist/fertility specialist (8)
- Other (10) ____________________
- I don't know (9)

If you weren't already seeing one, were you referred to a Perinatologist/Maternal Fetal Medicine (MFM) specialist IMMEDIATELY (within two days) following your TTTS diagnosis?

- Yes (1)
- No (2)
- I was already seeing a Perinatologist/Maternal Fetal Medicine (MFM) specialist when diagnosed (3)
- I don't know (4)

IMMEDIATELY following your TTTS diagnosis, how frequently were you receiving ultrasounds?

- Multiple times per day (1)
- Daily (2)
- Multiple times per week (3)
- Weekly (8)
- Bi-weekly (every other week) (4)
- Less than bi-weekly (every other week) (5)
- I was not receiving ultrasounds (6)
- I don't know (7)

Did you have a posterior or anterior placenta?

- Posterior (1)
- Anterior (2)
- I don't know (3)

Drag, in order from FIRST TO LAST the treatments that were OFFERED to you.

______ Bed rest (1)

______ Laser ablation surgery (2)

______ Selective reduction (3)

______ Protein therapy (4)

______ Amnioreduction (5)

______ Termination of the pregnancy (6)

______ Septostomy (7)

______ "Wait and see" monitoring (8)

______ Delivery (9)

______ No treatments offered (10)

Drag, in order from FIRST TO LAST the treatments that you USED.

______ Bed rest (1)

______ Laser ablation surgery (2)

______ Selective reduction (3)

______ Protein therapy (4)

______ Amnioreduction (5)

______ Termination of the pregnancy (6)

______ Septostomy (7)

______ "Wait and see" monitoring (8)

______ Delivery (9)

______ No treatments offered (10)

Do you feel that treatment options, and their repercussions, were explained thoroughly enough to you?

- Yes (1)
- No (2)
- I don't know (3)

Do you experience you own (maternal) side effects (e.g., physical, emotional and/or psychological) from the treatment choices you chose?

- Yes (1)
- No (2)
- I don't know (3)

If you experienced any of your own (maternal) side effects from the treatment choices you chose, please list them below (i.e. physical, emotional and/or psychological).

To what extent were you affected by anxiety (1 - not at all, 2 - a little, 3 - some, 4 - serious):

______PRIOR TO your TTTS pregnancy (1)

______DURING your TTTS pregnancy (2)

______AFTER your TTTS pregnancy (3)

To what extent were you affected by sadness/depression (1 - not at all, 2 - a little, 3 - some, 4 - serious):

______PRIOR TO your TTTS pregnancy (1)

______DURING your TTTS pregnancy (2)

______AFTER your TTTS pregnancy (3)

If you were diagnosed with a behavioral health condition PRIOR TO your TTTS pregnancy, what is the type of illness you experienced or may be experiencing?  Please select all that apply:

- Depression (2)
- Bipolar Disorder (3)
- Anxiety (4)
- Schizophrenia (5)
- Post-traumatic Stress Disorder (PTSD) (6)
- Attention Deficit Hyperactivity Disorder (ADHD) (7)
- Substance Abuse Condition (8)
- Eating Disorder (10)
- Other (9) ____________________
- I was not diagnosed with a behavioral health condition prior to my TTTS pregnancy (1)
- I do not want to say (12)

Did you experience a change in symptoms DURING your TTTS pregnancy?

If you were newly diagnosed with a behavioral health condition DURING your TTTS pregnancy, what is the type of illness you experienced or may be experiencing?  Please select all that apply:

- Depression (2)
- Bipolar Disorder (3)
- Anxiety (4)
- Schizophrenia (5)
- Post-traumatic Stress Disorder (PTSD) (6)
- Attention Deficit Hyperactivity Disorder (ADHD) (7)
- Substance Abuse Condition (8)
- Eating Disorder (10)
- Other (9) ____________________
- I was not diagnosed with a behavioral health condition during my TTTS pregnancy (1)
- I do not want to say (12)

Did you experience a change in symptoms AFTER your TTTS pregnancy?

If you were newly diagnosed with a behavioral health condition AFTER your TTTS pregnancy, what is the type of illness you experienced or may be experiencing?  Please select all that apply:

- Depression (2)
- Bipolar Disorder (3)
- Anxiety (4)
- Schizophrenia (5)
- Post-traumatic Stress Disorder (PTSD) (6)
- Attention Deficit Hyperactivity Disorder (ADHD) (7)
- Substance Abuse Condition (8)
- Eating Disorder (9)
- Other (10) ____________________
- I was not diagnosed with a behavioral health condition after my TTTS pregnancy (1)
- I do not want to say (11)

PRIOR TO your TTTS pregnancy, did you ever think about getting help for a mental health problem like depression,  anxiety, substance abuse, or other issue?

- Yes (1)
- No (2)

PRIOR TO your TTTS pregnancy, did you receive any type of behavioral health treatment? Select all that apply.

- Individual Counseling (1)
- Group Counseling (2)
- Formal Support Group (e.g., Alcoholics Anonymous) (3)
- Behavioral Health Medication, please specify (4) ____________________
- Hospitalization for behavioral health (5)
- Other, please specify (6) ____________________
- No, I did not receive any treatment (7)

DURING your TTTS pregnancy, did you ever think about getting help for a mental health problem like depression, anxiety, substance abuse, or other issue?

- Yes (1)
- No (2)

What would have been your preferred way to receive behavioral health services (e.g., counseling) DURING your TTTS pregnancy?  Rank order your preference by dragging items so 1 is your most preferred method and 3 is your least preferred method.

______ Talk to a counselor, social worker, or psychologist over the phone (1)

______ Talk to a counselor, social worker, or psychologist using an internet service like SKYPE (2)

______ Talk to a counselor, social worker, or psychologist in-person in their office (3)

DURING your TTTS pregnancy, did you receive any type of behavioral health treatment? Select all that apply.

- Individual Counseling (1)
- Group Counseling (2)
- Formal Support Group (e.g., Alcoholics Anonymous) (3)
- Behavioral Health Medication, please specify (4) ____________________
- Hospitalization for behavioral health (5)
- Other, please specify (7) ____________________
- No, I did not receive any treatment (6)
- I do not want to say (9)

If you received behavioral health treatment DURING your TTTS pregnancy, how useful were the services you received?

- Very Useless (1)
- Useless (2)
- Neutral (3)
- Useful (4)
- Very Useful (5)

If services were not useful, please explain why in the space provided below.

What, if anything, could have been done to help you receive behavioral health treatment DURING your pregnancy?

AFTER your TTTS pregnancy, did you ever think about getting help for a mental health problem like depression, anxiety, substance abuse, or other issue?

- Yes (1)
- No (2)
- I do not want to say (3)

What would have been your preferred way to receive behavioral health services such as counseling AFTER your TTTS pregnancy?  Rank order your preference by dragging items so 1 is your most preferred method and 3 is your least preferred method.

______ Talk to a counselor, social worker, or psychologist over the phone (1)

______ Talk to a counselor, social worker, or psychologist using an internet service like SKYPE (2)

______ Talk to a counselor, social worker, or psychologist in-person in their office (3)

AFTER your TTTS pregnancy, did you receive any type of behavioral health treatment? Select all that apply.

- Individual Counseling (1)
- Group Counseling (2)
- Formal Support Group (e.g., Alcoholics Anonymous) (3)
- Behavioral Health Medication, please specify (4) ____________________
- Hospitalization for behavioral health (5)
- No, I did not receive any treatment (6)
- I do not want to say (7)

If you received behavioral health treatment AFTER your TTTS pregnancy, how useful were the services you received?

- Very Useless (1)
- Useless (2)
- Neutral (3)
- Useful (4)
- Very Useful (5)

If services were not useful, please explain why in the space provided below.

What, if anything, could have been done to help you receive behavioral health treatment AFTER your pregnancy?

How important do you think it is for families to be offered the following services by healthcare providers DURING their TTTS pregnancy (1 – not at all important, 2 – very unimportant, 3 – neither important nor unimportant, 4 – very important, 5 – extremely important)?

______Individual counseling (1)

______Group counseling (2)

______Formal support group (e.g., formal meetings) (3)

______Informal support group (e.g., Facebook groups) (4)

______Behavioral health medication (5)

How important do you think it is for families to be offered the following services by healthcare providers AFTER their TTTS pregnancy (1 – not at all important, 2 – very unimportant, 3 – neither important nor unimportant, 4 – very important, 5 – extremely important)?

______Individual counseling (1)

______Group counseling (2)

______Formal support group (e.g., formal meetings) (3)

______Informal support group (e.g., Facebook groups) (4)

______Behavioral health medication (5)

Which did you experience?

- Double loss (1)
- Single survivor (2)
- Double survivors (3)
- Other (please specify) (4) ____________________
- I do not wish to say (5)

If you have two survivors, do you know which survivor is the donor twin and which survivor is the recipient twin?

- Yes (1)
- No (2)
- Not applicable (3)
- I do not wish to say (4)

If you experienced a single survivor, was your survivor the donor or recipient twin?

- Donor (1)
- Recipient (2)
- I don't know (3)
- I do not wish to say (4)

Did you experience your loss in utero or after giving birth? (You may select more than one)

- In utero loss (1)
- Loss quickly after birth (baby never went to NICU) (2)
- NICU loss (3)
- Baby was discharged from the hospital but passed away due to other causes prior to 12 months of age (4)
- Other (Please specify) (5) ____________________

Are your babies boys or girls?

- Boys (1)
- Girls (2)
- I don't know (3)

What were the birth weights of your babies?

Birth weight baby 1 (1)

Birth weight baby 2 (2)

Other (Please specify) (3)

Did your donor or recipient weigh MORE at birth?

- Donor (1)
- Recipient (2)
- Babies were the same weight (3)
- I don't know (4)

If you have one or more survivors, do they have any life-long health conditions that you believe to be a result of TTTS?

- Yes (1)
- No (2)
- I don't know (3)

Please list any PHYSICAL health conditions (for example, congential heart disease, kidney disease) affecting your survivors that you believe to be a result of TTTS.

DONOR (1)

RECIPIENT (2)

Please list any PHYSICAL health conditions (for example, congenital heart disease, kidney disease) affecting your survivors that you believe to be a result of TTTS.

Twin 1 (1)

Twin 2 (2)

Please list any PHYSICAL health conditions (for example, congenital heart disease, kidney disease) affecting your survivor that you believe to be a result of TTTS.

Please list any DEVELOPMENTAL health conditions (for example, developmental delay, developmental disability, cerebral palsy, autism, pervasive developmental disorder) affecting your survivors that you believe to be a result of TTTS.

DONOR (1)

RECIPIENT (2)

Please list any DEVELOPMENTAL health conditions (for example, developmental delay, developmental disability, cerebral palsy, autism, pervasive developmental disorder) affecting your survivors that you believe to be a result of TTTS.

Twin 1 (1)

Twin 2 (2)

Please list any DEVELOPMENTAL health conditions (for example, developmental delay, developmental disability, cerebral palsy, autism, pervasive developmental disorder) affecting your survivor that you believe to be a result of TTTS.

Please list any COGNITIVE conditions (for example, cognitive disability, learning disability, speech or language disorders) affecting your survivors that you believe to be a result of TTTS.

DONOR (1)

RECIPIENT (2)

Please list any COGNITIVE conditions (for example, cognitive disability, learning disability, speech or language disorders) affecting your survivors that you believe to be a result of TTTS.

Twin 1 (1)

Twin 2 (2)

Please list any COGNITIVE conditions (for example, cognitive disability, learning disability, speech or language disorders) affecting your survivor that you believe to be a result of TTTS.

Please list any OTHER conditions not already mentioned affecting your survivors that you believe to be a result of TTTS.

DONOR (1)

RECIPIENT (2)

Please list any OTHER conditions not already mentioned affecting your survivors that you believe to be a result of TTTS.

Twin 1 (1)

Twin 2 (2)

Please list any OTHER conditions not already mentioned affecting your survivor that you believe to be a result of TTTS.

In what gestational week did you deliver?

Did you deliver vaginally or via cesarean section? (You may select more than one)

- Vaginally (1)
- Cesarean section (2)
- I don't know (3)

If you had a vaginal delivery, was this by choice or would you have preferred a cesarean section delivery?

- Yes, the vaginal delivery was by choice. (1)
- No, the vaginal delivery was not by choice and I would have preferred a cesarean section. (2)
- I don't know (3)

Did you experience infertility prior to this pregnancy?

- Yes (1)
- No (2)
- I don't know (3)

Did you use the assistance of a reproductive endocrinologist or fertility specialist during the cycle in which this pregnancy was achieved?

- Yes (1)
- No (2)
- I don't know (3)

Did you use any of the following methods during the cycle in which this pregnancy was achieved? (You may select more than one)

- Ovulation inducing medication, like Clomid (1)
- Follicle stimulating medication, like Follistim or Menopur (2)
- Intra-uterine insemination (IUI) (3)
- In-vitro fertilization (IVF) (4)
- Other (please specify) (5) ____________________

If you used in-vitro fertilization (IVF) during the cycle in which this pregnancy was achieved, what type of process was used? (You may select more than one)

- Fresh transfer (1)
- Frozen transfer (2)
- 3-day transfer (3)
- 5-day blastocyst transfer (4)
- Intracytoplasmic sperm injection, or ICSI (5)
- Assisted embryo hatching, or AH (6)
- Other (7) ____________________

Were you informed about the possibility of having identical twins as a result of certain IVF techniques?

- Yes. Please specify which IVF techniques you were told could result in identical twins (1) ____________________
- No (2)
- I don't know (3)

Into whose care were you referred by your reproductive endocrinologist/fertility specialist?

- Midwife (1)
- Primary care obstetrician (2)
- Perinatologist/Maternal Fetal Medicine (MFM) specialist (3)
- Other (6) ____________________
- I was not referred to anyone by my reproductive endocrinologist/fertility specialist (4)
- I don't know (5)

Were any medical professionals a source of support or assistance for you during your TTTS experience?

- Yes (1)
- No (2)
- I don't know (3)

Please indicate which of the following individuals were a source of support or assistance (You may select more than one)

- Obstetrician (1)
- Perinatologist/Maternal Fetal Medicine (MFM) specialist (2)
- Nurse (3)
- Midwife (4)
- Nurse Practitioner (5)
- Physician Assistant (6)
- Ultrasound Tech (7)
- Reproductive endocrinologist/fertility specialist (8)
- Other (10) ____________________

Describe how the medical professional(s) was a source of support for you emotionally?

Describe how the medical professional(s) was a source of support for you medically?

While battling TTTS, did you make use of any of the following resources? (You may select more than one)

- Social networking sites like Facebook (1)
- Other online support groups (2)
- In-person support groups (3)
- The TTTS Foundation (4)
- The Fetal Health Foundation (5)
- Your own medical searches (PubMed, Google Scholar, etc.) (6)
- Other literature (7)
- Contacting other doctors (8)
- Other (9) ____________________
- I did not use any resources (10)

How was your relationship with your spouse or partner (1- Very good, 2- Good, 3-Neutral, 4-Poor, 5-Very Poor, 6-I did not have a spouse or partner):

______PRIOR TO your TTTS diagnosis? (1)

______DURING your TTTS experience? (2)

______in the DAYS immediately FOLLOWING delivery? (3)

______SIX MONTHS AFTER the conclusion of your pregnancy? (4)

Did you experience a divorce or permanent separation from your spouse of partner following your TTTS pregnancy?

- Yes (1)
- No (2)
- I did not have a spouse or partner (3)

Do you feel as anything related to your TTTS experience had significant impact on your divorce or permanent separation?

- Yes (1)
- No (2)
- I don't know (3)

Please explain why you believe your TTTS experience had a significant impact on your divorce or separation.

How were your relationships with your close family (excluding spouse or partner) (1- Very good, 2- Good, 3-Neutral, 4-Poor, 5-Very Poor, 6-I did not have close family):

______PRIOR TO your TTTS diagnosis? (1)

______DURING your TTTS experience? (2)

______in the DAYS immediately FOLLOWING delivery? (3)

______SIX MONTHS AFTER the conclusion of your pregnancy? (4)

How were your relationships with your close friends (1- Very good, 2- Good, 3-Neutral, 4-Poor, 5-Very Poor, 6-I did not have close friends):

______PRIOR TO your TTTS diagnosis? (1)

______DURING your TTTS experience? (2)

______in the DAYS immediately FOLLOWING delivery? (3)

______SIX MONTHS AFTER the conclusion of your pregnancy? (4)

Do you feel as if you received the best possible care you could have by your PRIMARY OBSTETRICIAN? Please describe, in detail, why yes or no.

Do you feel as if you received the best possible care you could have by your PERINATOLOGIST/MATERNAL FETAL MEDICINE (MFM) SPECIALIST? Please describe, in detail, why yes or no.

Do you feel as if you received the best possible care you could have by your REPRODUCTIVE ENDOCRINOLOGIST/FERTILITY SPECIALIST? Please describe, in detail, why yes or no.

Do you feel as if you received the best possible care you could have by your ANY OTHER MEMBERS OF YOUR PREGNANCY TEAM? Please describe, in detail, why yes or no.

Throughout your entire monochorionic-diamniotic pregnancy and TTTS experience, did you ever feel the need to advocate for additional care to your primary obstetrician (or other primary care providers)?

- Yes (1)
- No (2)
- I don't know (3)

If you answered yes to the previous question, please describe how you advocated and why.

Did your primary obstetrician (or other primary care providers) listen to your concerns and act accordingly? Please be specific and outline how each of your concerns was handled.

Please give any comments you feel are vital regarding your TTTS experience that were not collected within this survey.
